# A Polygenic Approach to Understanding Resilience to Peer Victimisation

**DOI:** 10.1007/s10519-021-10085-5

**Published:** 2021-10-11

**Authors:** Jessica M. Armitage, R. Adele H. Wang, Oliver S. P. Davis, Claire M. A. Haworth

**Affiliations:** 1 School of Psychological Science, University of Bristol, Bristol, BS8 1TU UK; 2 MRC Integrative Epidemiology Unit, University of Bristol, Bristol, BS8 2BN UK; 3 School of Economics, Finance and Management, University of Bristol, BS8 1TU Bristol, UK; 4 Department of Population Health Sciences, Bristol Medical School, University of Bristol, Bristol, BS8 1UD UK; 5 The Alan Turing Institute, British Library, London, NW1 2DB UK; 6 NIHR Biomedical Research Centre, The University Hospitals Bristol NHS Foundation Trust, University of Bristol, BS8 2BN Bristol, UK

**Keywords:** Polygenic scores, Victimisation, Resilience, Depression, Wellbeing, ALSPAC

## Abstract

Previous studies suggest an individual’s risk of depression following adversity may be moderated by their genetic liability. No study, however, has examined peer victimisation, an experience repeatedly associated with mental illness. We explore whether the negative mental health outcomes following victimisation can be partly attributed to genetic factors using polygenic scores for depression and wellbeing. Among participants from the Avon Longitudinal Study of Parents and Children (ALSPAC), we show that polygenic scores and peer victimisation are significant independent predictors of depressive symptoms (n=2268) and wellbeing (n=2299) in early adulthood. When testing for interaction effects, our results lead us to conclude that low mental health and wellbeing following peer victimisation is unlikely to be explained by a moderating effect of genetic factors, as indexed by current polygenic scores. Genetic profiling is therefore unlikely to be effective in identifying those more vulnerable to the effects of victimisation at present. The reasons why some go on to experience mental health problems following victimisation, while others remain resilient, requires further exploration, but our results rule out a major influence of current polygenic scores.

**Supplementary Information:**

The online version contains supplementary material available at 10.1007/s10519-021-10085-5.

## Introduction

Depression is a debilitating disorder and leading cause of worldwide disability (World Health Organization 2018). Heritability estimates for depression are around 30%–40% (Sullivan, Neale and Kendler 2000), highlighting a significant role for genetic factors. Genome-wide association studies (GWAS) into the genetic architecture of depression have revealed it is highly polygenic (Howard et al. 2019), meaning effects are driven by numerous genetic variants, each of small effect. These can be indexed by polygenic risk scores and used to estimate an individual’s genetic risk of depression (Lewis and Vassos 2020).

Polygenic scores are calculated using the weighted sum of independent risk alleles from a discovery GWAS (Dudbridge 2013). By summarising the combined effects of multiple genetic variants, polygenic scores provide a more accurate representation of the genetic risk of complex traits like depression compared to single candidate genes. Their utility is highlighted by research that has demonstrated an ability to predict disease status within both case-control studies (Howard et al. 2019) and population-based cohorts (Musliner et al. 2019). Current polygenic scores account for approximately 3% of the variance in depression (Howard et al. 2019), suggesting their main effects alone may have a negligible impact on the risk of depression. Researchers have therefore urged that studies consider interactions between genetic and environmental factors when investigating the etiology of psychiatric disorders (Assary et al. 2017).

Although genetic liability to a trait or disorder is fixed from birth, the risk is dynamic, meaning it may be altered by certain exposures (Haworth and Davis 2014). Diathesis-stress models propose that stress activates a predisposed vulnerability, or diathesis, which eventually transpires into sufficient conditions for disorder onset (Monroe and Simons 1991). Thus, the genetic risk of psychiatric disorders may be heightened by stressful environments. This genetically driven sensitivity towards environments can be empirically tested through gene-by-environment studies.

Gene-environment interaction (G×E) studies assess the extent to which psychiatric risk is influenced by genetic predispositions and environmental exposures. The presence of a G×E indicates that the influence of an environment is different for individuals with different genotypes (Ottman 1996). A G×E can also refer to the different outcomes of a genotype among individuals with differing environmental exposures (Ottman 1996). Studies using the G×E framework to test the diathesis-stress model of depression have shown that interactions between polygenic scores and negative life events predict an increased risk of depression (Colodro-Conde et al. 2018). Such findings imply a heightened risk for individuals with both genetic vulnerability to depression and negative life experiences.

Similar findings have been reported when exploring the specific impact of childhood trauma (Peyrot et al. 2014), although findings are conflicted. One study provided evidence of an inverse association and found that individuals subjected to childhood trauma are at greatest risk if they also have a low polygenic score for depression (Mullins et al. 2016). These findings were contrary to what was predicted, which was that polygenic scores would correlate with an increased risk. Such a finding is yet to repeated, with others reporting no interactions between polygenic risk and childhood trauma in predicting depression (Peyrot et al. 2018). A recent phenome-wide association study which investigated the association between depression polygenic scores and many environments, revealed varying interactive results depending on the age at which the trauma took place, with interactions identified using childhood trauma but not trauma that took place in adulthood (Shen et al. 2020a). No study to date has used the polygenic approach to consider experiences in adolescence, a critical period for the onset of mental health problems (Kessler et al. 2005), or the role of peer victimisation.

Peer victimisation refers to the experience in which an individual is repeatedly exposed to discomfort at the expense of another peer’s actions (Olweus 1994). It is a common occurrence in schools worldwide, with prevalence rates ranging from 8.6%–45.2% (Craig et al. 2009). Peer victimisation is deemed a major public health concern (Srabstein and Merrick 2013), associated with adverse outcomes across the lifespan, including depression (Arsenault 2017) and low wellbeing (Armitage et al. 2021). Not all individuals exposed to peer victimisation however, go on to develop problems with their mental health. Around 15% of individuals who report frequent victimisation during adolescence have a clinical diagnosis of depression by early adulthood (Bowes et al. 2015), suggesting many display resilience to its effects.

It is possible that some individuals are especially vulnerable to the effects of victimisation due to their heightened genetic risk. Heritability estimates for peer victimisation range from 65%–77% (Johansson et al. 2020; Veldkamp et al. 2019). Research has revealed that this genetic susceptibility may be explained by an increased genetic risk towards other traits and disorders, such as depression (Schoeler et al. 2019). This finding of a shared genetic liability has led some to propose that the negative outcomes of peer victimisation may largely reflect pre-existing vulnerabilities (Singham et al. 2017). This gene-environment correlation (rGE), whereby the risk of an individual experiencing an event is partly attributed to their genotype (Plomin et al. 1977), could account for both the increased risk of peer victimisation and the subsequent susceptibility to depression. Individuals more capable of displaying resilience after peer victimisation may therefore be those at a lower genetic risk to mental health problems.

One study investigating this theory attempted to predict outcomes of individuals following the death of a spouse using polygenic scores (Domingue et al. 2017). It was found that individuals with higher wellbeing polygenic scores showed significantly smaller increases in depressive symptoms than those with lower polygenic scores following the loss of a loved one. This study, however, did not investigate whether the polygenic scores moderated levels of wellbeing, but focused solely on buffering effects on depression. Wellbeing refers to feelings of satisfaction and happiness (Diener 2000) and is therefore more than the absence of a mental illness (Westerhof and Keyes 2010). Studies have shown overlapping but also distinct genetic and environmental factors associated with depression and wellbeing (Haworth et al. 2017), highlighting the need to consider both to attain a more complete understanding of resilient functioning.

## Current Study

The aim of the current study was to test the hypothesis that the relationship between peer victimisation in adolescence and mental health in early adulthood is moderated by an individual’s polygenic risk. We explore both depression and wellbeing outcomes to provide the first insight into whether genetic information can be used to target those more vulnerable to the effects of victimisation to help foster resilience.

## Methods

### Participants

Phenotypic and genotype data were taken from the Avon Longitudinal Study of Parents and Children (ALSPAC; Boyd et al. 2013), a prospective cohort based in the United Kingdom. Pregnant women residing in the former Avon area, with an expected delivery date between April 1991 and December 1992 were enrolled for the study (Fraser et al. 2013). The initial cohort consisted of 14,062 live births but has since increased to 14,901 children who were alive after one year with further recruitment (Northstone et al. 2019). Data gathered from 22 years and onwards were collected and managed using REDCap electronic data capture tools hosted at the University of Bristol (Harris et al. 2009). Please note that the study website contains details of all the data that is available through a fully searchable data dictionary and variable search tool (http://www.bristol.ac.uk/alspac/researchers/our-data/). Further information relating to genotyping can be found in the supplementary material.

Our study involved individuals who completed the victimisation assessment at 13 years (*n*=6527) and provided genotype data (*n=*4829). Of these, we used data from 2268 individuals who also completed the assessment for depressive symptoms at 23 years, and from 2299 individuals who completed the wellbeing questionnaire at 23 years. Individuals with genotype data were more likely to be female and less likely to be of a non-white ethnicity, consistent with previous genetic studies (Mullins et al. 2016). However, victimisation scores did not significantly differ between those with and without genotype information, or between those with missing data on the mental health outcomes (Supplementary Table S1). Ethical approval for our study was obtained from the ALSPAC Ethics and Law Committee and the Local Research Ethics Committees. Informed consent for the use of data collected via questionnaires and clinics was obtained from participants following the recommendations of the ALSPAC Ethics and Law Committee at the time.

## Measures

### Peer Victimisation

Peer victimisation was measured at 13 years using the previously validated Bullying and Friendship Interview Schedule (Wolke et al. 2001). Participants responded to nine statements relating to direct and indirect experiences of victimisation within the last six months. Direct victimisation is characterised by physical or verbal acts of aggression, while indirect relates to experiences of social exclusion. Adolescents responded based on the frequency of these experiences (0=Never, 1=Seldom, 2=Frequently, 3=Very Frequently). Correlations between the direct and indirect items were moderate (r=0.52), therefore scores from all items were summed. Scores ranged from 0–25 (M= 1.81, SD=2.75), with 0 representing those who had never been bullied. Owing to high amounts of positive skew (skew=2.4), victimisation scores were log transformed. This reduced the skew to 0.72. Main analyses were carried out using the log-transformed scores and repeated using the untransformed scores. Results using the untransformed scores are presented in the supplementary materials (see Table S2).

### Mental Health

At 23 years, depressive symptoms were assessed using the Short Mood and Feelings Questionnaire (SMFQ) (Angold et al. 1995). The MFQ has proven a reliable and valid measure of depression in both clinical and non-clinical samples (Daviss et al. 2006). The shortened version represents a 13-item scale derived from the 33-item Moods and Feelings Questionnaire (MFQ) which aims to capture the presence of depression symptoms within the last two weeks (Costello and Angold 1988). Overall scores on the sMFQ range from 0 to 26, with a score of 12 or above indicative of depression. Scores in the current sample had a mean of 6.64 (SD=5.82) and a skew greater than 1 (skew=1.15). This was adjusted for in the regression analyses using a negative binomial model, as described below.

Wellbeing was assessed for the first time at 23 years using the Warwick-Edinburgh Mental Well-Being Scale (WEMWBS) (Tennant et al. 2007). Although other wellbeing measures were available, we chose this scale because of its ability to capture affective and cognitive aspects, as well as overall psychological functioning (Tennant et al. 2007). WEMWBS is also widely used within public health and policy and has proven reliability across populations in Europe (López et al. 2013). The scale comprises of 14 items relating to experiences over the last two weeks. Individuals choose from a 5-point Likert scale that best describes their experience. Items are scored positively and summed to produce a minimum score of 14 and a maximum score of 70. Scores in the current sample had a mean average of 49.17 (SD=8.74) and a slight positive skew (skew=− 0.41). However, because this skew value was below 1, analyses were conducting using a standard linear regression.

### Polygenic Scores

Polygenic scores for depression were created in PRSice (http://prsice.info, Euesden, Lewis, and O'Reilly 2015) using publicly available summary statistics from the largest GWAS to date of major depression (Howard et al. 2019). This GWAS meta-analysed data from the three largest GWASs of depression (Howard et al. 2018; Hyde et al 2016; Wray et al. 2018). The studies each used a different diagnostic instrument to assess depression, with one based on self-reported help-seeking behaviour, another on self-declared clinical depression, and the third used clinically obtained reports. Despite this, strong genetic correlations (>0.85) were identified between the three, suggesting an overlap in the underlying genetic architecture.

For comparative purposes, polygenic scores associated with wellbeing were also investigated. These were also created in PRSice using summary data from the multivariate genome-wide-association meta-analysis (GWAMA) of wellbeing (Baselmans et al. 2019). We used data from the N-weighted multivariate GWAMA (N-GWAMA) to generate overall wellbeing-polygenic scores. The N-GWAMA is a novel method that was introduced by the authors to test for a unitary effect of SNPs on four related traits: life satisfaction, positive affect, neuroticism, and depressive symptoms. The authors collectively refer to these as the well-being spectrum (Baselmans et al. 2019).

Both the depression-polygenic scores and wellbeing-polygenic scores were generated by combining the number of risk alleles present for each SNP (0, 1, or 2), weighted by their effect estimates as reported in the original discovery GWAS. ALSPAC is an independent sample and was not included in either of the discovery GWASs. Each SNP was used to construct a polygenic score in the ALSPAC cohort using best guess imputation genotypes. SNPs with a minor allele frequency (MAF) <1% and an imputation quality score <0.8 have been removed. Clumping was carried out to remove SNPs in linkage disequilibrium (LD) based on an r-squared threshold of 0.10 within a 500kb window. This was to align with previous procedures using both the depression (Howard et al. 2019) and the wellbeing GWAS (Baselmans et al. 2019). Polygenic scores were initially calculated using *p*-value thresholds of 5×10^−8^, 1×10^−6^, 1×10^−4^, 0.001, 0.01, 0.1, 0.2, 0.3, 0.4, 0.5 and 1. The number of SNPs included were 70, 159, 1000, 2197, 12924, 62678, 99128, 127673, 150781, 169733 and 192822 respectively for the depression-polygenic scores and 198, 418, 1628, 4009, 12381, 38939, 55626, 68192, 78160, 86119 and 107155 for the wellbeing-polygenic scores. All polygenic scores were z-standardised to a mean of 0 and standard deviation of 1 to facilitate interpretability (Lewis and Vassos 2017). Correlations between the depression and wellbeing polygenic scores, as well as with peer victimisation and the mental health measures can be found in Table 1.

**Table 1:** Correlations between study variables

Correlation matrix									
Variables	1	2	3	4	5	6	7	8	9
1. Peer victimisation (log)	1	− .11*** (− .16, − .07)	.17*** (.13, .21)	.02 (− .02, .06)	.04 (− .00, .08)	.06*** (.02, .10)	.07*** (.02, − .09)	− .05* (− .09, − .01)	− .05* (− .09, − .01)
2. Mental wellbeing		1	− .69*** (− .71, − .67)	− .05*** (− .09, − .01)	− .03 (− .07, − .01)	− .10*** (− .14, − .06)	− .11*** (− .15, − .07)	.08*** (.04, .12)	.14*** (.10, .18)
3. Depressive symptoms			1	.09*** (.05, .14)	**.001** (− .04, .04)	.12*** (.08, .16)	.13*** (.08, .16)	− .08*** (− .12, − .04)	− .15*** (− .19, − .11)
4. Sex				.1	.02 (− .03, .06)	.03 (− .02, .07)	.03 (− .008, .07)	− .03 (− .07, .01)	− .02 (− .06, .02)
5. Depression-polygenic scores (P^T^ 5 × 10^–8^)					1	.18*** (.13, .22)	.17*** (.13, .21)	− .62*** (− .64, − .59)	− .34*** (− .38, − .31)
6. Depression-polygenic scores (P^T^ 0.1)						1	.94*** (.93, .95)	-.18*** (− .22, − .14)	− .35*** (− .38, − .31)
7. Depression-polygenic scores (P^T^ 0.2)							1	− .16*** (− .20, − .12)	− .34*** (− .38, − .30)
8. Wellbeing-polygenic scores (P^T^ 5 × 10^–8^)								1	.51*** (.47, .54)
9. Wellbeing-polygenic scores (P^T^ 0.001)									1

Text in bold denotes p < 0.05

P^T^ = p-value threshold of the polygenic score. n = 2232. Mental wellbeing and depressive symptoms were assessed at 23 years and sex was coded as 0 = Male and 1 = Female

^***^p < 0.001, **p < 0.01, *p < 0.05

## Statistical Analyses

The main effects of the polygenic scores on depression and wellbeing were first examined using linear regressions. These models controlled for sex and the first two genetic principal components (PCs) to reduce possible subtle confounding by population stratification, as per previous studies (Mullins et al. 2016). The number of PCs to include depends largely on the variation within the sample (Anderson et al. 2019). Given that our cohort comprised of individuals of white European ancestry who were from a single region, 2 PCs were deemed sufficient to control for population stratification. We ran models exploring all SNP-association thresholds to inform the polygenic scores that had the highest incremental R^2^ value. This was calculated by separately regressing the depressive symptom and wellbeing outcomes onto sex and the two PCs, and then comparing models to those that included the polygenic scores. This is common practice for selecting which scores to use for subsequent analyses (Anderson et al. 2019). We then re-test the main effects of the polygenic-scores with the highest variance while also adjusting for the log-transformed victimisation scores. This allowed us to determine the main and independent effects of both victimisation and polygenic risk. We subsequently test for a potential gene-environment correlation (rGE) by exploring associations between the polygenic scores and victimisation. Studying both forms of gene-environment interplay (rGE and G×E) is vital as omission of either could lead to an overestimation of environmental effects (Eaves et al. 2003).

To investigate whether the polygenic scores moderate the wellbeing and depressive symptoms of individuals exposed to victimisation, we subsequently ran regression models that included an interaction term (victimisation by the polygenic scores). These initially used the genome-wide significant polygenic scores (*p* < 5×10^-8^) and then further analyses explored other significance thresholds. These thresholds were chosen to reflect the largest main effects for each polygenic score (see Supplementary Figure S1). All analyses controlled for the main effects of the polygenic scores and victimisation, as recommended when modelling interaction terms (Greenland and Pearce 2015), as well as sex and the first two PCs to correct for population stratification. To adjust for the potential effects of covariates on the interaction, we included adjustments for all covariate x polygenic score and covariate x victimisation interactions, as previously recommended (Keller 2014).

All analyses predicting wellbeing were run using standard linear regression, while analyses predicting depressive symptoms used negative binomial regression to address the negative skew. Negative binomial regression models were chosen over the standard Poisson model as the Poisson regression assumes identical parameters for the mean and variance, this was not the case for our depressive symptoms measure (M=6.64, σ²=34.8). We used the ‘MASS’ package (Venables and Ripley 2002) in R Studio version 4.5.0 (R Core Team 2021) to carry out the negative binomial regression models and the ‘rsq’ package (Zhang 2018) to generate R-squared estimates. To control for the probability of making a Type I error on multiple comparisons, Benjamini-Hochberg False Discovery Rate (FDR; Benjamini and Hochberg 1995) was used. This approach allows for the non-independence of repeated tests and works by controlling for the expected proportion of incorrect rejections at a designated value. This was set as α = 0.05 for the current study.

## Results

### Participant Characteristics

Of the individuals who completed the wellbeing and depressive symptom assessments, 54% scored above 0 on the victimisation scale, indicating some experience of victimisation in adolescence. Of these, 12.4% experienced frequent victimisation, defined as scoring 1 SD above the mean, as per previous research (Stadler et al. 2010). Among individuals who provided complete data and who reported some experience of victimisation, 18.7% had clinically relevant symptoms of depression compared to 12% of individuals who did not experience victimisation. Wellbeing scores averaged 48.53 (range 14–70) among those who experienced some victimisation, with scores averaging 50.04 (range 17-70) among those never victimised. Depressive symptoms and wellbeing were both predicted by sex, with females more likely to report increased depressive symptoms (ß=.172, SE=.04, p<0.001) and reduced wellbeing (ß=− 1.08, SE=.38, p<0.001).

### Main Effect Analyses

When exploring associations between our outcome variables and the depression-polygenic scores, we find that the direction of effects was as predicted; all scores were positively associated with depressive symptoms, and negatively associated with wellbeing, with estimates typically stronger using more liberal thresholds (see Table S3). The depression-polygenic scores with a p-value threshold of 0.1 explained the most variance in depressive symptoms (incremental R^2^ = 1.43%), while the p-value threshold of 0.2 explained the most variance in wellbeing (incremental R^2^ = 1.21%). When investigating the wellbeing-polygenic scores, associations were also in the expected direction, with positive associations found with wellbeing, and negative associations with depressive symptoms (Table S4). The p-value threshold of 0.001 explained the most variance in both wellbeing (incremental R^2^ = 2.09%) and depressive symptoms (incremental R^2^ = 2.11%).

When investigating the main effects of the polygenic scores after accounting for victimisation, findings revealed highly similar results for both the depression- and wellbeing-polygenic scores (see Table S5). The main effects of victimisation on our outcome variables also remained largely the same after accounting for polygenic risk (Table S5). Such findings suggest that both victimisation and the polygenic scores are independent predictors of depressive symptoms and wellbeing. To explore a possible rGE between victimisation and the polygenic scores, we subsequently explored associations between the two. Findings revealed that some of the polygenic scores were associated with victimisation (Table 2). However, the depression-polygenic scores explained just 0.42% of the variance in victimisation, while the wellbeing-polygenic scores accounted for up to 0.45% of the variance. Correlations between the polygenic scores and victimisation were also low, reaching *r*=0.06 between victimisation and the depression-polygenic scores, and *r*=− 0.07 with the wellbeing-polygenic scores. We therefore believe it unlikely that any G×E findings will be largely attributable to a rGE.

**Table 2:** Main effects of polygenic scores on victimisation at 13 years (i.e. gene-environment correlation)

P^T^	Main effects of depression-polygenic scores^a^			Main effects of wellbeing-polygenic scores^a^		
	Beta (CI)	P	ΔR^2^	Beta (CI)	P	ΔR^2^
5 × 10^8^	0.095 (-0.009, 0.198)	0.073	0.14%	− 0.125 (− 0.230, − 0.020)	**0.020**	0.24%
1 × 10^6^	0.124 (0.019, 0.228)	**0.020**	0.24%	− 0.129 (− 0.235, − 0.023)	**0.017**	0.26%
1 × 10^4^	0.159 (0.053, 0.265)	**0.003**	0.38%	− 0.171 (− 0.276, − 0.065)	**0.002**	0.45%
0.001	0.123 (0.017, 0.229)	**0.022**	0.23%	− 0.116 (− 0.222, − 0.010)	**0.032**	0.21%
0.01	0.161 (0.058, 0.263)	**0.002**	0.42%	− 0.128 (− 0.232, − 0.023)	**0.017**	0.26%
0.1	0.143 (0.041, 0.245)	**0.006**	0.34%	− 0.097 (− 0.202, 0.008)	0.070	0.15%
0.2	0.161 (0.057, 0.262)	**0.002**	0.41%	− 0.077 (− 0.182, 0.028)	0.151	0.09%
0.3	0.163 (0.059, 0.266)	**0.002**	0.42%	− 0.065 (− 0.170, 0.041)	0.228	0.07%
0.4	0.162 (0.058, 0.265)	**0.002**	0.42%	− 0.057 (− 0.163, 0.049)	0.292	0.05%
0.5	0.159 (0.056, 0.263)	**0.003**	0.41%	− 0.059 (− 0.165, 0.047)	0.274	0.05%
1	0.157 (0.053, 0.260)	**0.003**	0.39%	− 0.058 (− 0.163, 0.048)	0.284	0.05%

Text in bold denotes p < 0.05

P^T^ = p-value threshold of the polygenic score. ΔR^2^ represents the incremental R^2^. This is the percentage of variance explained by the polygenic risk score. The incremental R^2^ was calculated by regressing victimisation on sex and the first two principal components of ancestry, and then including the polygenic scores and comparing the variance explained

^a^Linear regression models were used to separately investigate the main effects of the depression-polygenic scores and wellbeing-polygenic scores on victimisation among individuals with complete victimisation and mental health data (n = 2232). To account for possible effects of population stratification, models controlled for two principal components and sex

### Interaction Analyses to Test for G×E Effects

**Fig. 1:**
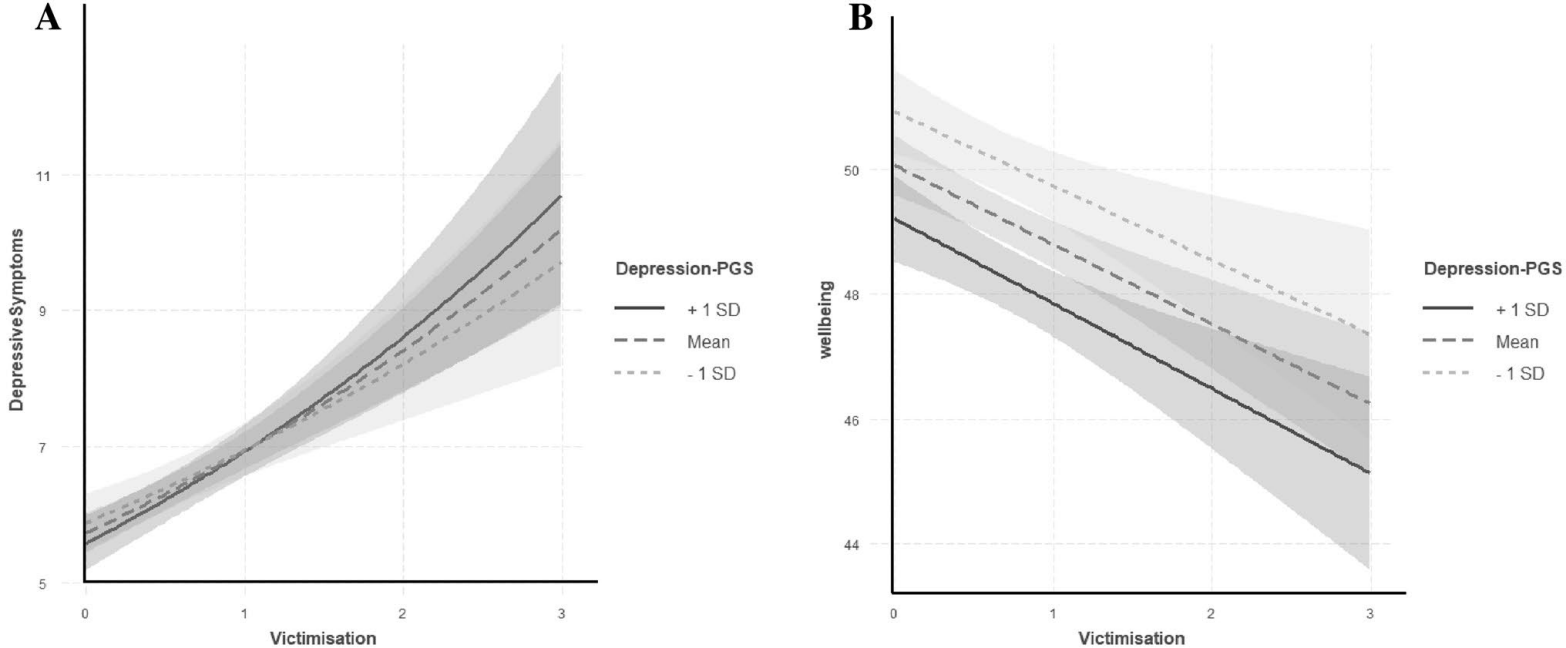
Interactive effects of log-transformed victimisation scores and the depression-polygenic scores (PGS) (P-value thereshold=5×10^8^) on depressive symptoms and wellbeing. **A** demonstrates no differences in depressive symptoms at α= 0.05 among victims with varying polygenic scores. **B** provides some evidence of an effect of polygenic risk towards depression on wellbeing scores, with those reporting higher victimisation scores and a PGS 1 SD above the mean more likely to report lower wellbeing. This difference in wellbeing scores corresponded to p=0.056

No interactions were found between either the depression- or wellbeing-polygenic scores and victimisation in predicting either depressive symptoms or wellbeing (Table 3). When using depression-polygenic scores based on the genome-wide significant threshold, findings revealed a borderline interaction effect (*p=* 0.056) when predicting wellbeing (Fig. 1). While plots of these findings suggest that having a lower polygenic risk to depression could prove protective to wellbeing for those exposed to more victimisation, the difference in wellbeing scores between individuals at a high polygenic risk was not significantly different. This was also the case for individuals exposed to no victimisation whose wellbeing scores were largely similar among those at a low or high genetic risk to depression. It is interesting to note that when entered as an interaction term, victimisation remained associated with an increased risk of depressive symptoms but was no longer predictive of wellbeing. This is likely due to the significant negative interactions that were observed between victimisation and sex in all models predicting wellbeing but not depressive symptoms. This suggests that associations between victimisation and wellbeing are heavily driven by sex; with female victims more likely to experience reductions in their wellbeing compared to males. Such findings demonstrate the importance of appropriate control over confounding variables as such effects can lead to misinterpretations of interactive effects (Keller 2014).

Analyses using the untransformed victimisation scores revealed similar results, with confidence intervals that overlapped with those using the transformed scores. (Table S2). These analyses revealed a significant interaction term between the depression-polygenic scores and victimisation in predicting wellbeing, however such findings were not robust after correction for multiple testing. For completeness, results from the other polygenic score thresholds can be found in the supplementary (see Table S6). Findings were highly consistent across different polygenic thresholds.

**Table 3:** Impact of log-transformed victimisation scores at age 13, polygenic scores, and their interaction on depressive symptoms and wellbeing at 23 years

	Polygenic Scores		Victimisation		Interaction			
	β (95% C.I.)	P value	β (95% C.I)	P value	β (95% C.I)	P value	R^2^	ΔR^2^
Impact on depressive symptoms^a^								
Depression-PGS								
P^T^ = 5 × 10^–8^	− 0.048 (− 0.129, 0.033)	0.259	0.188 (0.084, 0.293)	4.0× 10^–4^†	0.024 (− 0.022, 0.070)	0.318	3.1%	0.1%
P^T^ = 0.1	0.100 (0.017, 0.184)	0.017	0.177 (0.073, 0.281)	8.0× 10^–4^†	− 0.027 (− 0.074, 0.021)	0.271	4.3%	1.3%
Wellbein-PGS								
P^T^ = 5 × 10^–8^	− 0.035 (− 0.121, 0.050)	0.418	0.181 (0.077, 0.286)	6.0× 10^–4^†	0.005 (− 0.044, 0.054)	0.844	3.6%	0.6%
P^T^ = 0.001	− 0.068 (− 0.154, 0.018)	0.124	0.176 (0.072, 0.281)	7.9× 10^–4^†	− 0.003 (− 0.050, 0.045)	0.911	5.2%	2.2%
Impact on wellbeing^b^								
Depression-PGS								
P^T^ = 5 × 10^–8^	0.091 (− 0.702, 0.885)	0.821	− 0.014 (− 0.979, 1.01)	0.977	− 0.452 (− 0.916, 0.012)	0.056	2.5%	0.9%
P^T^ = 0.2	− 0.365 (− 1.17, 0.443)	0.376	− 0.022 (− 0.969, 1.01)	0.965	− 0.085 (− 0.554, 0.384)	0.722	3.5%	1.7%
Wellbeing-PGS								
P^T^ = 5 × 10^–8^	0.232 (− 0.583, 1.05)	0.577	− 0.045 (− 1.04, − 0.949)	0.930	− 0.014 (− 0.497, 0.468)	0.953	2.9%	1.2%
P^T^ = 0.001	0.074 (− 0.743, 0.890)	0.860	0.004 (− 0.980, 0.989)	0.993	0.311 (− 1.53, 0.776)	0.189	4.6%	2.9%

Each row represents a separate multiple regression of either depressive symptoms or wellbeing predicted by the polygenic scores, victimisation, and the gene-environment interaction

PGS = Polygenic score. P^T^ = p value threshold of the polygenic score. R^2^ is the variance accounted for by the main and interactive effects of victimisation and the polygenic scores, as well as the covariates. ΔR^2^ represents the incremental R^2^. †FDR

^a^Negative binomial regression models were used to investigate the main and interactive effects of the polygenic scores and victimisation on depressive symptoms aged 23 (n = 2268).

^b^ Linear regression models were used to investigate the main and interactive effects of the polygenic scores and victimisation on wellbeing aged 23 (n = 2299)

## Discussion

We consider for the first time whether the increased risk of mental illness and low wellbeing following victimisation can be partly attributed to genetic factors using polygenic scores for depression and wellbeing. Polygenic scores derived from the depression GWAS of adult samples explained up to 1.60% of the variance in depressive symptoms in our emerging adulthood sample, while wellbeing-polygenic scores explained up to 2.56% of the variance in wellbeing. These estimates are slightly higher than previous reports for both depression (Mullins et al. 2016) and wellbeing (Okbay et al. 2016), reflecting the increase in power gained from using larger and more recent meta-analyses of GWAS data. Overall, we report significant independent effects of the polygenic scores and peer victimisation in predicting the risk of depressive symptoms and wellbeing, but no clear interaction effects.

Few studies have considered the underlying paths driving resilience to the effects of peer victimisation. Those that have explored possible pathways have focused on estimating the role of genes and environments in influencing protective factors (Bowes, Maughan, Caspi, Moffitt and Arseneault 2010). By expanding this research to explore the impact of genetic liabilities to specific traits, our study offers novel insight into the potential moderating role of genetic risk. Findings suggest that the risk for poorer mental health among victims is not significantly different for those with varying polygenic risk to depression and wellbeing. Such findings are not consistent with diathesis-stress models of depression (Colodre et al. 2018) which would hypothesise that the mental health of victims varies according to their polygenic risk.

Our findings are also not consistent with the presence of a strong gene-environment correlation. To investigate gene-environment correlations, we explored associations between the polygenic scores and victimisation. Findings revealed that while some of the polygenic scores predicted the risk of peer victimisation (Table 2), the variance explained was small. This suggests that the risk of experiencing peer victimisation cannot be largely accounted for by an increased genetic risk to depression or wellbeing, as encapsulated by the current polygenic scores. Such findings may explain the absence of strong moderating effects of the polygenic scores. It is possible that the relationship between victimisation and mental health might be one in which genetic factors have a negligible effect. Similar conclusions were drawn from a twin study which reported that although pre-existing vulnerabilities may increase the likelihood of victimisation, they do not solely explain the increased risk of psychopathology among victims (Schaefer et al. 2018). This was based on the finding that victims experienced increased mental health problems independent of their early-life emotional problems, family background, and genetic risk (Schafer et al. 2018). The authors interpreted this as suggestive of a direct impact of victimisation on mental illness.

Our results lead us to a similar conclusion and allow us to also conclude that having a low polygenic risk to depression, or a high polygenic score for wellbeing, does not clearly reduce the risk of peer victimisation, or significantly buffer against mental illness to predict resilience to victimisation. Equally, findings suggest that having a higher genetic risk towards depression and low wellbeing does not heighten the risk of poor mental following peer victimisation. These weak moderating effects of genetic risk factors reflect previous findings from a meta-analysis of G×E studies (Peyrot et al. 2018). This revealed that the risk of depression among individuals exposed to childhood trauma is unlikely to be attributable to a moderation of genome-wide genetic effects. While our study results do not completely rule out the presence of a G×E, they suggest that using the current polygenic scores, there are no meaningful moderating effects on the mental health of individuals exposed to peer victimisation. Our study had 80% power to detect the current interactive effects at a = 0.05 (see Supplementary), therefore it is likely that larger discovery GWASs will be necessary to detect more subtle interactive effects.

It is important that when interpreting the absence of main and moderating effects of the polygenic scores that researchers are mindful of what the polygenic scores represent. Polygenic scores only include additive genetic effects and therefore do not represent the full genetic loading for a trait or disorder. In addition to this, while polygenic scores do capture information from the environment (Lewis et al. 2020), such as through environmentally mediated parental genetic effects, it is unclear from the current design to what degree this occurred. While the goal of the present study was not to study the aetiology of depression or wellbeing but possible pathways from peer victimisation to resilience, future research using polygenic scores within a family-based design could provide further insight into potential gene-by-environment correlations (Selzam et al. 2019).

Our study has both strengths and limitations. We are the first to explore predictors of both depression and wellbeing, and represent the only study to use polygenic scores for both depression and wellbeing. While our polygenic scores for wellbeing were derived from a GWAS that included measures of depression, we still identify moderate but not identical (rG~0.62) correlations between our depression- and wellbeing-polygenic scores. This suggests that they each capture independent genetic effects, reinforcing the need to expand current research focused solely on depression. Our findings also encourage more careful consideration of the thresholds used to derive the polygenic scores. Previous studies have tended to use arbitrary SNP *p*-value thresholds, which is a likely contribution to the lack of consistency within the literature. We present findings using the genome-wide significance cut-off to ensure polygenic scores were specific to the trait of interest and less likely to reflect noise. It is important to note however, that these scores did not have main effects at a = 0.05 on our outcome variables. We therefore supplement analyses using polygenic scores that captured the most variance in our outcome variables. All analyses were adjusted for multiple testing, as previously advised when exploring possible SNP-thresholds for analyses (Anderson et al. 2019).

One potential limitation of our methods for creating the polygenic scores is that we selected SNPs based on their main effects. These may not be the same variants that are involved in G×E. The generalisability of our findings must also be considered in relation to the discovery GWASs used to construct our polygenic scores. The extent to which participants from the original GWASs were victimised is unknown. It is therefore not possible to make deductions about the relative impact on our results. Both GWASs also included large samples from the UKBiobank. Participants from the UKBiobank are on average healthier than the general population, are more likely to be of white European decent, and are less likely to come from socioeconomically deprived areas (Fry et al. 2017). Assessments of mental health in these groups may therefore have less variation than the general population. Findings have shown, however, that prevalence rates for mental health disorders are largely similar across members from the UKBiobank and the general population (Davis et al. 2018). Recent research has also revealed that polygenic scores derived from these studies can be used to predict psychiatric disorders among individuals from different ancestral and cultural backgrounds (Shen et al. 2020b). It was noted, however, that the predictive ability of these polygenic scores is reduced compared to predictions among European samples. Nevertheless, these findings offer promising scope for the future of polygenic scores. Further research should now seek to include larger and more diverse samples in GWASs. This will be crucial to reducing the potential for health disparities that may arise from further polygenic research (Martin et al. 2019), and will prove key to ensuring any potential benefits offer improvements for both European and non-European populations (Lewis and Vassos 2020).

Overall, our study provides a unique approach to the study of resilience following peer victimisation. While we find no strong evidence that the mental health of victims is moderated by their polygenic risk to depression or wellbeing, further research using larger and more highly powered GWAS samples is necessary to rule it out. This will help detect more subtle G×E effects to clarify whether genetic profiling could be used to identify those more vulnerable to the effects of victimisation. Such findings could have clinical relevance and allow us to understand more about the reasons some go on to experience mental health problems, while others remain resilient following peer victimisation.

## Supplementary Information

Below is the link to the electronic supplementary material.

Supplementary file1 (DOCX 159 kb)

## Data Availability

The data analysed in the current study is not publicly available. This is because the informed consent obtained from participants in ALSPAC does not allow data to be made freely available through any third party maintained public repository. Data used for this submission, however, can be made available on request to the ALSPAC Executive. Please refer to the ALSPAC data management plan which describes the policy regarding data sharing. This is through a system of managed open access. Full instructions for applying for data access can be found here: http://www.bristol.ac.uk/alspac/researchers/access/. The ALSPAC study website contains details of all the data that are available (http://www.bristol.ac.uk/alspac/researchers/our-data/).
