# Supplementary material for: A Polygenic Approach to Understanding Resilience to Peer Victimisation
Source: Behav Genet. 2021 Oct 11;52(1):1–12. doi: 10.1007/s10519-021-10085-5 (PMC8770424; doi:10.1007/s10519-021-10085-5)

**Supplementary material**

**A polygenic approach to understanding resilience to peer victimisation**

Jessica M. Armitage^a,b^, R.Adele H. Wang^c^, Oliver S.P. Davis^b,d,e,f^ and Claire M.A. Haworth^a,e,f^

Author affiliations:

^a^ School of Psychological Science, University of Bristol, Bristol, BS8 1TU, United Kingdom.

^b^ MRC Integrative Epidemiology Unit, University of Bristol, Bristol, BS8 2BN, United Kingdom.

^c^ School of Economics, Finance and Management, University of Bristol, BS8 1TU, United Kingdom.

^d^ Department of Population Health Sciences, Bristol Medical School, University of Bristol, Bristol, BS8 1UD, United Kingdom.

^e^ The Alan Turing Institute, British Library, London, NW1 2DB, UK.

^f^ NIHR Biomedical Research Centre at the University Hospitals Bristol NHS Foundation Trust and the University of Bristol, Bristol, BS8 2BN, United Kingdom.

**Table of contents**

**Supplementary Methods**

1. Genotyping of ALSPAC sample
2. Power analyses

**Supplementary Tables**

1. **Table S1:** Sample characteristics of those in current study compared to those missing
2. **Table S2:** Impact of untransformed victimisation scores, polygenic scores, and their interaction on depressive symptoms and wellbeing at 23 years
3. **Table S3:** Association between the depression-polygenic scores and depressive symptoms and wellbeing at 23 years
4. **Table S4:** Association between the wellbeing-polygenic scores and depressive symptoms and wellbeing at 23 years
5. **Table S5:** Main effects of polygenic scores and log-transformed victimisation scores on depressive symptoms and wellbeing at 23 years
6. **Table S6:** Impact of log-transformed victimisation scores, polygenic scores, and their interaction on depressive symptoms and wellbeing at 23 years using the other polygenic thresholds
7. **Figure S1:** Proportion of variance in depressive symptoms and wellbeing explained by the polygenic scores at each p-value threshold

**Supplementary methods**

**Genotyping of ALSPAC sample**

9912 children in ALSPAC were genotyped using the Illumina HumanHap550 Quad Array platform (run at the SangerInstitute, Cambridge UK and Laboratory Corporation of America, Burlington, USA). These data were subject to quality control measures in which SNPs were excluded if they met the following: a minor allele frequency (MAF) of <0.01, a call rate of <0.95, an individual call rate of <0.97, info <0.80 or if there was evidence of violations of Hardy-Weinberg equilibrium (HWE, p<5x10^-7^). Individual participants were also removed if there were incorrect sex assignments, minimal or excessive heterozygosity, disproportionate missingness (>3%), evidence of cryptic relatedness (proportion of identity by descent >0.125), insufficient sample replication and non-European ancestry. Individuals not of European descent were detected by a multidimensional scaling analysis seeded with Hapmap II (release 22) individuals. After these exclusions, and following imputation of the data, performed using Phase 1 of the 1000 Genomes reference panel from the Impute2 reference data repository, genotype data were available for 8,237 children.

**Power analyses**

Power calculations to detect main effects of the polygenic scores were carried out using the ‘avengeme’ package in R studio (Dudbridge, 2013). These were calculated for the depression and wellbeing polygenic scores using the thresholds that explained the most variance when assessing main effects. Power calculations for the depression-polygenic scores were based on the GWAS discovery sample of 807,553, which explained up to 3.2% of the variance in depression. Using the depression-polygenic scores at a threshold of 0.1, our study had 80% power to detect 0.9% of the phenotypic variance. For wellbeing, the discovery sample was 2,370,390. Polygenic scores generated from this sample explained up to 1.10% of the variance in wellbeing. When using the wellbeing-polygenic scores at a threshold of 0.001, our study had 80% power to detect 0.8% of the phenotype variance.

Power analyses for the interaction linear regression models were carried out using G*Power 3.1 (Faul, Erdfelder, Buchner & Lang, 2009) with α = 0.05 and 12 predictors (polygenic risk score, victimisation, sex, principal component 1, principal component 2, polygenic score*victimisation, sex*victimisation, sex*polygenic score, principal component1*victimisation, principal component2*victimisation, principal component1*polygenic score, principal component2*polygenic score). Using the maximum sample of the current study (n=2299), analyses had 80% power to detect effects that explain 0.82% of the phenotypic variance.

| **Table S1:** Sample characteristics of those in current study compared to those missing | | | | | | | |
| --- | --- | --- | --- | --- | --- | --- | --- |
|  | **Victimisation responders (n=6527) ^a^** | **Victimisation and genotype**  **(n=4829) ^b^** | **MFQ responders**  **(n=2268) ^c^** | **Missing MFQ responders (n=2561) ^d^** | **Wellbeing responders (n=2299) ^e^** | **Missing wellbeing responders (n=2530) ^f^** | **ALSPAC Sample (n=15443) ^g^** |
| Female (%) | 48.8 | 51.4 | 63.9 | 40.4 | 63.6 | 40.4 | 48.8 |
| Victimised at least once (%) | 53.7 | 54.1 | 53.7 | 54.5 | 53.8 | 54.4 | - |
| Overall victimisation score, M(SD) | 1.82 (2.76) | 1.81 (2.69) | 1.73 (2.52) | 1.88 (2.84) | 1.73 (2.53) | 1.87 (2.84) | - |
| Non-white (%) | 3.9 | 0.2 | 0.3 | 0.2 | 0.3 | 0.2 | 5.0 |
| Parents own car (%) | 94.8 | 95.3 | 96.6 | 94.2 | 96.5 | 94.2 | 90.8 |
| Parents married (%) | 83.7 | 84.9 | 86.9 | 83.0 | 86.8 | 83.1 | 79.5 |
| Mother was homeowner (%) | 85.1 | 86.5 | 89.2 | 84.1 | 89.2 | 84.1 | 77.1 |
| Mother has University degree (%) | 17.2 | 18.9 | 23.7 | 14.5 | 23.6 | 14.1 | 13.7 |
| *Note:*  ^a^ Individuals who completed the victimisation assessment at 13 years  ^b^ Individuals with genotype data who completed the victimisation assessment at 13 years  ^c^ Individuals with genotype data who completed the victimisation assessment at 13 years and the MFQ at 23 years  ^d^ Individuals with genotype data who completed the victimisation assessment at 13 years but not the MFQ at 23 years  ^e^ Individuals with genotype data who completed the victimisation assessment at 13 years and the WEMWBS at 23 years  ^f^ Individuals with genotype data who completed the victimisation assessment at 13 years but not the WEMWBS at 23 years  ^g^ Core singleton ALSPAC sample. | | | | | | | |

**Supplementary Tables**

| **Table S2:** Impact of untransformed victimisation scores, polygenic scores, and their interaction on depressive symptoms and wellbeing at 23 years | | | | | | | | | | | | |
| --- | --- | --- | --- | --- | --- | --- | --- | --- | --- | --- | --- | --- |
|  |  | **Polygenic scores** | | **Victimisation** | | | | | **Interaction** | | | |
|  |  | **β (95% C.I.)** | **P value** | |  | **β (95% C.I)** | **P value** |  | **β (95% C.I)** | **P value** | **R^2^** | **ΔR^2^** |
| **Depressive symptoms**^a^ | | |  | |  |  |  |  |  |  |  |  |
| ***Depression-polygenic scores*** | | |  | |  |  |  |  |  |  |  |  |
| P^T^=5x10^8^ |  | -0.038 (-0.117, 0.040) | 0.348 | |  | 0.045 (0.013, 0.078) | **0.005** |  | 0.005 (-0.009, 0.019) | 0.466 | 2.7% | 0.3% |
| P^T^=0.1 |  | 0.092 (0.011, 0.174) | **0.022** | |  | 0.043 (0.010, 0.075) | **0.007** |  | -0.006 (-0.021, 0.008) | 0.370 | 3.8% | 0.8% |
| ***Wellbeing-polygenic scores*** | | |  | |  |  |  |  |  |  |  |  |
| P^T^=5x10^8^ |  | -0.037 (-0.120, 0.045) | 0.374 | |  | 0.043 (0.012, 0.076) | **0.006** |  | 0.001 (-0.014, 0.017) | 0.864 | 3.2% | 0.2% |
| P^T^=0.001 |  | -0.108 (-0.192, -0.024) | **0.011** | |  | 0.041 (0.009, 0.073) | **0.009** |  | 0.001 (-0.015, 0.013) | 0.850 | 4.9% | 1.9% |
| **Wellbeing**^b^ |  |  | | | | | | | | |  |  |
| ***Depression-polygenic scores*** | | |  | |  |  |  |  |  |  |  |  |
| P^T^=5x10^8^ |  | 0.080 (-0.688, 0.849) | 0.838 | |  | -0.056 (-0.242, 0.354) | 0.713 |  | -0.164 (-0.303, -0.026) | **0.020** | 2.6% | 0.9% |
| P^T^=0.2 |  | -0.329 (-1.11, 0.451) | 0.409 | |  | -0.054 (-0.244, 0.352) | 0.721 |  | -0.044 (-0.184, 0.095) | 0.532 | 3.5% | 1.8% |
| ***Wellbeing-polygenic scores*** | | |  | |  |  |  |  |  |  |  |  |
| P^T^=5x10^8^ |  | 0.149 (-0.638, 0.936) | 0.711 | |  | -0.018 (-0.279, 0.315) | 0.905 |  | 0.043 (-0.110, 0.195) | 0.582 | 2.9% | 1.3% |
| P^T^=0.001 |  | 0.384 (-0.400, 1.17) | 0.337 | |  | -0.030 (-0.264, 0.324) | 0.841 |  | 0.108 (-0.022, 0.239) | 0.104 | 4.4% | 2.8% |
| *Note*:  Findings presented in this table have not been adjusted for multiple testing.  P^T^ = p value threshold of the polygenic score. R^2^ is the variance accounted for by the main and interactive effects of victimisation and the polygenic scores, as well as the covariates. ΔR^2^ represents the incremental R^2^. This is the percentage of variance explained by the addition of the polygenic scores. The ΔR^2^ was calculated by regressing the outcome on victimisation and the covariates, and then including the interaction term with the polygenic scores and comparing the variance explained.  ^a^ Negative binomial regression models were used to investigate the main and interactive effects of the polygenic scores and victimisation on depressive symptoms aged 23 (n=2268). **^b^** Linear regression models were used to investigate the main and interactive effects of the polygenic scores and victimisation on wellbeing aged 23 (n=2299). | | | | | | | | | | | | |

| **Table S3:** Association between the depression-polygenic scores and depressive symptoms and wellbeing at 23 years | | | | | | | | | | |
| --- | --- | --- | --- | --- | --- | --- | --- | --- | --- | --- |
| **P^T^** | |  | **Main effects on depressive symptoms**^a^ | | |  | **Main effects on wellbeing^b^** | | | |
|  |  | **β** **(95% C.I.)** | | **P value** | **ΔR^2^** |  | | **β (95% C.I)** | **P value** | **ΔR^2^** |
| 5x10^8^ |  | 0.007 (-0.037, 0.035) | | .971 | 0.00% |  | | -0.271 (-0.625, 0.082) | 0.133 | 0.10% |
| 1x10^6^ |  | 0.028 (-0.008, 0.065) | | 0.133 | 0.13% |  | | -0.314 (-0.671, 0.042) | 0.084 | 0.13% |
| 1x10^4^ |  | 0.072 (0.034, 0.110) | | **1.8 ^x^ 10^-4^** | 0.52% |  | | -0.567 (-0.927, -0.207) | **0.002** | 0.41% |
| 0.001 |  | 0.086 (0.048, 0.123) | | **7.0 ^x^ 10^-6^** | 0.80% |  | | -0.741 (-1.10, -0.382) | **5.4 ^x^ 10^-5^** | 0.70% |
| 0.01 |  | 0.103 (0.067, 0.139) | | **2.3 ^x^ 10^-8^** | 1.34% |  | | -0.884 (-1.23, -0.534) | **8.1 ^x^ 10^-7^** | 1.05% |
| 0.1 |  | 0.105 (0.069, 0.140) | | **1.2 ^x^ 10^-8^** | 1.43% |  | | -0.912 (-1.26, -0.565) | **2.9 ^x^ 10^-7^** | 1.14% |
| 0.2 |  | 0.110 (0.073, 0.146) | | **4.9 ^x^ 10^-9^** | 1.38% |  | | -0.954 (-1.31, -0.600) | **1.4 ^x^ 10^-7^** | 1.21% |
| 0.3 |  | 0.110 (0.073, 0.146) | | **4.1 ^x^ 10^-9^** | 1.38% |  | | -0.921 (-1.27, -0.567) | **3.5 ^x^ 10^-7^** | 1.12% |
| 0.4 |  | 0.111 (0.074, 0.147) | | **3.2 ^x^ 10^-9^** | 1.42% |  | | -0.953 (-1.31, -0.600) | **1.3 ^x^ 10^-7^** | 1.20% |
| 0.5 |  | 0.107 (0.071, 0.144) | | **9.1 ^x^ 10^-9^** | 1.36% |  | | -0.953 (-1.31, -0.599) | **1.3 ^x^ 10^-7^** | 1.20% |
| 1 |  | 0.101 (0.065, 0.138) | | **6.2 ^x^ 10^-8^** | 1.20% |  | | -0.935 (-1.29, -0.581) | **2.4 ^x^ 10^-7^** | 1.15% |
| *Note:*  P^T^ = p-value threshold of the polygenic score. ΔR^2^ represents the incremental R^2^. This is the percentage of variance explained by the polygenic risk score. The incremental R^2^ was calculated by regressing the outcome on sex and the first two principal components of ancestry, and then including the polygenic scores and comparing the variance explained.  ^a^ Negative binomial regression models were used to investigate associations between the polygenic scores and depressive symptoms at 23 years (n=2268) **^b^** Linear regression models were used to investigate associations between the polygenic scores and wellbeing aged 23 (n=2299). To account for possible effects of population stratification, all models controlled for two principal components and sex. | | | | | | | | | | |

| **Table S4:** Association between the wellbeing-polygenic scores and depressive symptoms and wellbeing at 23 years | | | | | | | | | | |
| --- | --- | --- | --- | --- | --- | --- | --- | --- | --- | --- |
| **P^T^** | |  | **Main effects on depressive symptoms**^a^ | | |  | **Main effects on wellbeing^b^** | | | |
|  |  | **β** **(95% C.I.)** | | **P value** | **ΔR^2^** |  | | **β (95% C.I)** | **P value** | **ΔR^2^** |
| 5x10^8^ |  | -0.069 (-0.106, -0.032) | | **2.6 ^x^ 10^-4^** | 0.63% |  | | 0.671 (0.313, 1.03) | **2.4 ^x^ 10^-4^** | 0.58% |
| 1x10^6^ |  | -0.097 (-0.134, -0.061) | | **3.6 ^x^ 10^-7^** | 1.19% |  | | 0.850 (0.490, 1.21) | **3.9 ^x^ 10^-6^** | 0.92% |
| 1x10^4^ |  | -0.118 (-0.155, -0.081) | | **4.4 ^x^ 10^-10^** | 1.69% |  | | 1.08 (0.719, 1.43) | **3.9 ^x^ 10^-9^** | 1.49% |
| 0.001 |  | -0.134 (-0.172, -0.097) | | **1.7 ^x^ 10^-12^** | 2.11% |  | | 1.28 (0.992, 1.64) | **3.1 ^x^ 10^-12^** | 2.09% |
| 0.01 |  | -0.137 (-0.174, -0.100) | | **3.4 ^x^ 10^-13^** | 2.10% |  | | 1.25 (0.899, 1.61) | **4.7 ^x^ 10^-12^** | 2.06% |
| 0.1 |  | -0.129 (-0.166, -0.092) | | **6.9 ^x^ 10^-12^** | 1.87% |  | | 1.24 (0.882, 1.59) | **1.2 ^x^ 10^-11^** | 1.97% |
| 0.2 |  | -0.128 (-0.165, -0.091) | | **1.1 ^x^ 10^-11^** | 1.90% |  | | 1.23 (0.873, 1.59) | **1.9 ^x^ 10^-11^** | 1.94% |
| 0.3 |  | -0.124 (-0.161, -0.086) | | **5.6 ^x^ 10^-11^** | 1.80% |  | | 1.21 (0.891, 1.56) | **3.8 ^x^ 10^-11^** | 1.88% |
| 0.4 |  | -0.128 (-0.165, -0.091) | | **1.6 ^x^ 10^-11^** | 1.88% |  | | 1.24 (0.880, 1.60) | **1.6 ^x^ 10^-11^** | 1.95% |
| 0.5 |  | -0.129 (-0.166, -0.092) | | **9.1 ^x^ 10^-11^** | 1.93% |  | | 1.25 (0.894, 1.61) | **9.7 ^x^ 10^-12^** | 1.99% |
| 1 |  | -0.128 (-0.165, -0.091) | | **1.3 ^x^ 10^-11^** | 1.89% |  | | 1.24 (0.877, 1.59) | **1.7 ^x^ 10^-11^** | 1.95% |
| *Note:*  P^T^ = p-value threshold of the polygenic score. ΔR^2^ represents the incremental R^2^. This is the percentage of variance explained by the polygenic risk score. The incremental R^2^ was calculated by regressing the outcome on sex and the first two principal components of ancestry, and then including the polygenic scores and comparing the variance explained.  ^a^ Negative binomial regression models were used to investigate associations between the polygenic scores and depressive symptoms at 23 years (n=2268). **^b^** Linear regression models were used to investigate associations between the polygenic scores and wellbeing aged 23 (n=2299). To account for possible effects of population stratification, all models controlled for two principal components and sex. | | | | | | | | | | |

| **Table S5:** Main effects of polygenic scores and log-transformed victimisation scores on depressive symptoms and wellbeing at 23 years | | | | | | | | | |  |
| --- | --- | --- | --- | --- | --- | --- | --- | --- | --- | --- |
|  |  | **Main effects on depressive symptoms** ^a^ | | **Main effects on wellbeing**^b^ | | | | | | |
| **Main effects** |  | **β (95% C.I.)** | **P value** | |  | | **β (95% C.I)** | **P value** |  | |
| Victimisation (unadjusted) |  | 0.193 (0.144, 0.241) | **8.2 ^x^ 10^-15^** | | |  | -1.31 (-1.79, -0.846) | **4.9 ^x^ 10^-8^** |  | |
| Victimisation (adjusted for dep-PRS, P^T^=5x10^8^) |  | 0.193 (0.145, 0.242) | **7.2 ^x^ 10^-15^** | | |  | -1.31 (-1.78, -0.834) | **6.6 ^x^ 10^-8^** |  | |
| Victimisation (adjusted for dep-PRS, P^T^=0.1) |  | 0.187 (0.139, 0.236) | **3.3 ^x^ 10^-14^** | | |  | -1.26 (-1.73, -0.788) | **1.7 ^x^ 10^-7^** |  | |
| Victimisation (adjusted for wellbeing-PRS, P^T^=5x10^8^) |  | 0.189 (0.140, 0.237) | **2.6 ^x^ 10^-14^** | | |  | -1.28 (-1.75, -0.810) | **1.1 ^x^ 10^-8^** |  | |
| Victimisation (adjusted for wellbeing-PRS, P^T^=0.001) |  | 0.183 (0.135, 0.232) | **9.1 ^x^ 10^-13^** | | |  | -1.25 (-1.72, -0.780) | **1.9 ^x^ 10^-7^** |  | |
| ***Depression-polygenic scores*** |  |  |  | | |  |  |  |  | |
| P^T^=5x10^8^ (unadjusted) |  | 0.000 (-0.035, 0.037) | 0.966 | | |  | -0.276 (-0.630, 0.077) | 0.126 |  | |
| P^T^=5x10^8^ (adjusted for victimisation) |  | -0.008 (-0.043, 0.028) | 0.683 | | |  | -0.241 (-0.593, 0.111) | 0.179 |  | |
| P^T^=0.1 (unadjusted) |  | 0.105 (0.069, 0.141) | **1.2 ^x^ 10^-8^** | | |  | -0.926 (-1.27, -0.578) | **1.9 ^x^ 10^-7^** |  | |
| P^T^=0.1 (adjusted for victimisation) |  | 0.099 (0.064, 0.134) | **5.9 ^x^ 10^-8^** | | |  | -0.880 (-1.23, -0.534) | **6.5 ^x^ 10^-7^** |  | |
| ***Wellbeing-polygenic scores*** | | |  | | |  |  |  |  | |
| P^T^=5x10^8^ (unadjusted) |  | -0.073 (-0.110, -0.035) | **1.4 ^x^ 10^-4^** | | |  | 0.685 (0.327, 1.04) | **1.7 ^x^ 10^-4^** |  | |
| P^T^=5x10^8^ (adjusted for victimisation) |  | -0.067 (-0.102, -0.029) | **5.0 ^x^ 10^-4^** | | |  | 0.643 (0.287, 0.999) | **4.0 ^x^ 10^-4^** |  | |
| P^T^=0.001 (unadjusted) |  | -0.136 (-0.174, -0.099) | **1.1 ^x^ 10^-12^** | | |  | 1.29 (0.937, 1.65) | **1.7 ^x^ 10^-12^** |  | |
| P^T^=0.001 (adjusted for victimisation) |  | -0.128 (-0.165, -0.091) | **1.1 ^x^ 10^-11^** | | |  | 1.25 (0.898, 1.61) | **6.5 ^x^ 10^-12^** |  | |
| *Note:*  P^T^ = p-value threshold of the polygenic score.  ^a^ Negative binomial regression models were used to investigate the main effects of the polygenic scores and victimisation on depressive symptoms aged 23 (n=2268). **^b^** Linear regression models were used to investigate the main effects of the polygenic scores and victimisation on wellbeing aged 23 (n=2299). | | | | | | | | | |  |

| **Table S6:** Impact of log-transformed victimisation scores, polygenic scores, and their interaction on depressive symptoms and wellbeing at 23 years using the other polygenic thresholds | | | | | | | | | | | | |
| --- | --- | --- | --- | --- | --- | --- | --- | --- | --- | --- | --- | --- |
|  |  | **Polygenic scores** | | **Victimisation** | | | | | **Interaction** | | | |
|  |  | **β (95% C.I.)** | **P value** | |  | **β (95% C.I)** | **P value** |  | **β (95% C.I)** | **P value** | **R^2^** | **ΔR^2^** |
| **Depressive symptoms**^a^ | | |  | |  |  |  |  |  |  |  |  |
| ***Depression-polygenic scores*** | | |  | |  |  |  |  |  |  |  |  |
| P^T^=5x10^8^ |  | -0.048 (-0.129, 0.033) | 0.259 | |  | 0.188 (0.084, 0.293) | **4.0 ^x^ 10^-4^** |  | 0.024 (-0.022, 0.070) | 0.318 | 3.1% | 0.1% |
| P^T^=5x10^6^ |  | -0.014 (-0.096, 0.068) | 0.742 | |  | 0.185 (0.081, 0.290) | **0.005** |  | 0.012 (-0.034, 0.058) | 0.618 | 3.2% | 0.2% |
| P^T^=5x10^4^ |  | 0.058 (-0.027, 0.143) | 0.181 | |  | 0.178 (0.074, 0.284) | **7.6 ^x^ 10^-4^** |  | 0.008 (-0.041, 0.058) | 0.733 | 3.5% | 0.4% |
| P^T^=0.001 |  | 0.096 (0.013, 0.179) | **0.025** | |  | 0.174 (0.070, 0.278) | **0.001** |  | -0.004 (-0.048, 0.048) | 0.999 | 3.8% | 0.8% |
| P^T^=0.01 |  | 0.078 (-0.003, 0.158) | 0.060 | |  | 0.174 (0.070, 0.278) | **9.8 ^x^ 10^-4^** |  | -0.032 (-0.079, 0.014) | 0.172 | 4.4% | 1.4% |
| P^T^=0.1 |  | 0.100 (0.017, 0.184) | **0.017** | |  | 0.177 (0.073, 0.281) | **8.0 ^x^ 10^-4^** |  | -0.027 (-0.074, 0.021) | 0.271 | 4.3% | 1.3% |
| P^T^=0.2 |  | 0.097 (0.011, 0.184) | **0.023** | |  | 0.178 (0.074, 0.282) | **7.0 ^x^ 10^-4^** |  | -0.022 (-0.071, 0.026) | 0.362 | 4.2% | 1.2% |
| P^T^=0.3 |  | 0.103 (0.017, 0.189) | **0.015** | |  | 0.176 (0.073, 0.281) | **8.0 ^x^ 10^-4^** |  | -0.022 (-0.070, 0.027) | 0.381 | 4.2% | 1.2% |
| P^T^=0.4 |  | 0.099 (0.013, 0.186) | **0.021** | |  | 0.176 (0.072, 0.280) | **8.6 ^x^ 10^-4^** |  | -0.018 (-0.067, 0.030) | 0.455 | 4.3% | 1.3% |
| P^T^=0.5 |  | 0.100 (0.014, 0.186) | **0.020** | |  | 0.176 (0.073, 0.281) | **8.0 ^x^ 10^-4^** |  | -0.022 (-0.071, 0.026) | 0.366 | 4.2% | 1.2% |
| P^T^=1 |  | 0.095 (0.008, 0.181) | **0.028** | |  | 0.176 (0.073, 0.281) | **8.3 ^x^ 10^-4^** |  | -0.021 (-0.069, 0.028) | 0.409 | 4.1% | 1.1% |
| ***Wellbeing-polygenic scores*** | | |  | |  |  |  |  |  |  |  |  |
| P^T^=5x10^8^ |  | -0.035 (-0.121, 0.050) | 0.418 | |  | 0.181 (0.077, 0.286) | **6.0 ^x^ 10^-4^** |  | 0.005 (-0.044, 0.054) | 0.844 | 3.6% | 0.6% |
| P^T^=5x10^6^ |  | -0.078 (-0.164, 0.007) | 0.071 | |  | 0.179 (0.075, 0.284) | **6.6 ^x^ 10^-4^** |  | 0.017 (-0.030, 0.063) | 0.498 | 4.1% | 1.1% |
| P^T^=5x10^4^ |  | -0.069 (-0.156, 0.017) | 0.116 | |  | 0.176 (0.072, 0.281) | **8.6 ^x^ 10^-4^** |  | 0.006 (-0.040, 0.052) | 0.810 | 4.7% | 1.7% |
| P^T^=0.001 |  | -0.068 (-0.154, 0.018) | 0.124 | |  | 0.176 (0.072, 0.281) | **7.9 ^x^ 10^-4^** |  | -0.003 (-0.050, 0.045) | 0.911 | 5.2% | 2.2% |
| P^T^=0.01 |  | -0.101 (-0.188, -0.014) | **0.022** | |  | 0.170 (0.066, 0.275) | **0.001** |  | -0.006 (-0.054, 0.042) | 0.805 | 5.1% | 2.1% |
| P^T^=0.1 |  | -0.096 (-0.180, -0.011) | **0.027** | |  | 0.171 (0.067, 0.275) | **0.001** |  | 0.010 (-0.039, 0.060) | 0.679 | 4.9% | 1.9% |
| P^T^=0.2 |  | -0.108 (-0.194, -0.022) | **0.014** | |  | 0.170 (0.067, 0.274) | **0.001** |  | 0.011 (-0.039, 0.060) | 0.673 | 4.9% | 1.9% |
| P^T^=0.3 |  | -0.101 (-0.187, -0.015) | **0.022** | |  | 0.170 (0.067, 0.274) | **0.001** |  | 0.002 (-0.046, 0.051) | 0.924 | 4.8% | 1.8% |
| P^T^=0.4 |  | -0.108 (-0.194, -0.022) | **0.015** | |  | 0.170 (0.067, 0.274) | **0.001** |  | -0.007 (-0.050, 0.049) | 0.977 | 4.9% | 1.9% |
| P^T^=0.5 |  | -0.113 (-0.199, -0.027) | **0.011** | |  | 0.169 (0.065, 0.272) | **0.001** |  | -0.003 (-0.052, 0.046) | 0.908 | 4.9% | 1.9% |
| P^T^=1 |  | -0.110 (-0.196, -0.025) | **0.013** | |  | 0.168 (0.065, 0.272) | **0.001** |  | -0.005 (-0.054, 0.045) | 0.856 | 4.9% | 1.9% |
| **Wellbeing**^b^ |  |  | | | | | | | | |  |  |
| ***Depression-polygenic scores*** | | |  | |  |  |  |  |  |  |  |  |
| P^T^=5x10^8^ |  | 0.091 (-0.702, 0.885) | 0.821 | |  | -0.014 (-0.979, 1.01) | 0.977 |  | -0.452 (-0.916, 0.012) | 0.056 | 2.5% | 0.9% |
| P^T^=5x10^6^ |  | 0.174 (-0.623, 0.972) | 0.668 | |  | -0.052 (-1.05, 0.944) | 0.919 |  | -0.314 (-0.779, 0.150) | 0.185 | 2.4% | 0.8% |
| P^T^=5x10^4^ |  | 0.116 (-0.693, 0.926) | 0.778 | |  | -0.061 (-1.06, 0.935) | 0.904 |  | -0.367 (-0.832, 0.098) | 0.121 | 2.7% | 1.1% |
| P^T^=0.001 |  | -0.069 (-0.876, 0.737) | 0.866 | |  | 0.028 (-0.964, 1.02) | 0.955 |  | -0.536 (-1.00, -0.069) | **0.024** | 3.2% | 1.5% |
| P^T^=0.01 |  | -0.255 (-1.04, 0.527) | 0.522 | |  | 0.029 (-0.964, 1.02) | 0.954 |  | 0.089 (-0.365, 0.544) | 0.700 | 3.4% | 1.7% |
| P^T^=0.1 |  | -0.443 (-1.23, 0.347) | 0.271 | |  | 0.026 (-0.966, 1.02) | 0.959 |  | 0.070 (-0.392, 0.532) | 0.766 | 3.4% | 1.7% |
| P^T^=0.2 |  | -0.365 (-1.17, 0.443) | 0.376 | |  | 0.022 (-0.969, 1.01) | 0.965 |  | -0.085 (-0.554, 0.384) | 0.722 | 3.4% | 1.7% |
| P^T^=0.3 |  | -0.347 (-1.15, 0.457) | 0.397 | |  | 0.026 (-0.966, 1.02) | 0.958 |  | -0.119 (-0.590, 0.351) | 0.619 | 3.3% | 1.7% |
| P^T^=0.4 |  | -0.292 (-1.10, 0.518) | 0.479 | |  | 0.032 (-0.959, 1.02) | 0.949 |  | -0.156 (-0.628, 0.315) | 0.516 | 3.4% | 1.8% |
| P^T^=0.5 |  | -0.307 (-1.12, 0.503) | 0.457 | |  | 0.014 (-0.976, 1.00) | 0.977 |  | -0.090 (-0.563, 0.381) | 0.705 | 3.4% | 1.8% |
| P^T^=1 |  | -0.255 (-1.07, 0.556) | 0.537 | |  | 0.016 (-0.975, 1.01) | 0.975 |  | -0.129 (-0.604, 0.346) | 0.594 | 3.4% | 1.7% |
| ***Wellbeing-polygenic scores*** | | |  | |  |  |  |  |  |  |  |  |
| P^T^=5x10^8^ |  | 0.232 (-0.583, 1.05) | 0.577 | |  | -0.045 (-1.04, -0.949) | 0.930 |  | -0.014 (-0.497, 0.468) | 0.953 | 2.9% | 1.2% |
| P^T^=5x10^6^ |  | 0.122 (-0.686, 0.931) | 0.766 | |  | -0.079 (-1.07, 0.913) | 0.876 |  | 0.048 (-0.420, 0.516) | 0.840 | 3.3% | 1.6% |
| P^T^=5x10^4^ |  | -0.003 (-0.826, 0.819) | 0.994 | |  | -0.019 (-1.01, 0.972) | 0.971 |  | 0.374 (-0.082, 0.830) | 0.108 | 3.8% | 2.2% |
| P^T^=0.001 |  | 0.074 (-0.743, 0.890) | 0.860 | |  | 0.004 (-0.980, 0.989) | 0.993 |  | 0.311 (-1.53, 0.776) | 0.189 | 4.6% | 2.9% |
| P^T^=0.01 |  | 0.358 (-0.457, 1.17) | 0.389 | |  | 0.003 (-0.984, 0.985) | 0.996 |  | 0.312 (-0.152, 0.778) | 0.187 | 4.3% | 2.7% |
| P^T^=0.1 |  | 0.690 (-0.125, 1.50) | 0.097 | |  | 0.100 (-0.885, 1.09) | 0.841 |  | 0.166 (-0.313, -0.645) | 0.496 | 4.2% | 2.6% |
| P^T^=0.2 |  | 0.611 (-0.217, 1.44) | 0.148 | |  | 0.103 (-0.882, 1.09) | 0.838 |  | 0.201 (-0.280, 0.682) | 0.413 | 4.2% | 2.6% |
| P^T^=0.3 |  | 0.645 (-0.186, 1.48) | 0.128 | |  | 0.099 (-0.886, 1.08) | 0.844 |  | 0.219 (-0.262, 0.699) | 0.372 | 4.2% | 2.5% |
| P^T^=0.4 |  | 0.657 (-0.178, 1.49) | 0.123 | |  | 0.097 (-0.887, 1.08) | 0.847 |  | 0.261 (-0.221, 0.744) | 0.288 | 4.2% | 2.6% |
| P^T^=0.5 |  | 0.686 (-0.149, 1.52) | 0.107 | |  | 0.106 (-0.877, 1.09) | 0.832 |  | 0.285 (-0.197, 0.767) | 0.246 | 4.3% | 2.6% |
| P^T^=1 |  | 0.657 (-0.172, 1.49) | 0.120 | |  | 0.107 (-0.877, 1.09) | 0.831 |  | 0.288 (-0.193, 0.769) | 0.241 | 4.2% | 2.6% |
| *Note:*  Findings presented in this table have not been adjusted for multiple testing.  P^T^ = p value threshold of the polygenic score. R^2^ is the variance accounted for by the main and interactive effects of victimisation and the polygenic scores, as well as the covariates. ΔR^2^ represents the incremental R^2^. This is the percentage of variance explained by the addition of the polygenic scores. The ΔR^2^ was calculated by regressing the outcome on victimisation and the covariates, and then including the interaction term with the polygenic scores and comparing the variance explained.  ^a^ Negative binomial regression models were used to investigate the main and interactive effects of the polygenic scores and victimisation on depressive symptoms aged 23 (n=2268). **^b^** Linear regression models were used to investigate the main and interactive effects of the polygenic scores and victimisation on wellbeing aged 23 (n=2299) | | | | | | | | | | | | |

***Supplementary Figure S1:*** *Proportion of variance in depressive symptoms and wellbeing explained by the polygenic scores at each p-value threshold*


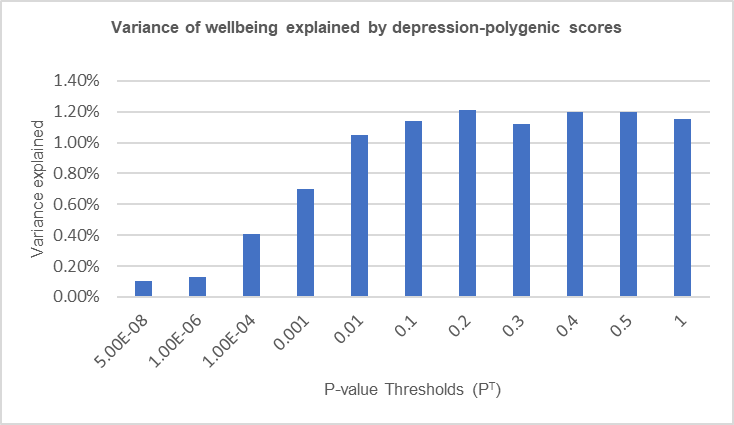

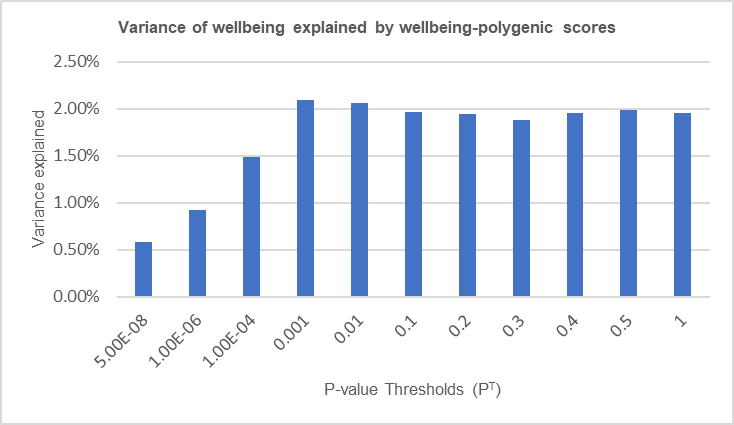

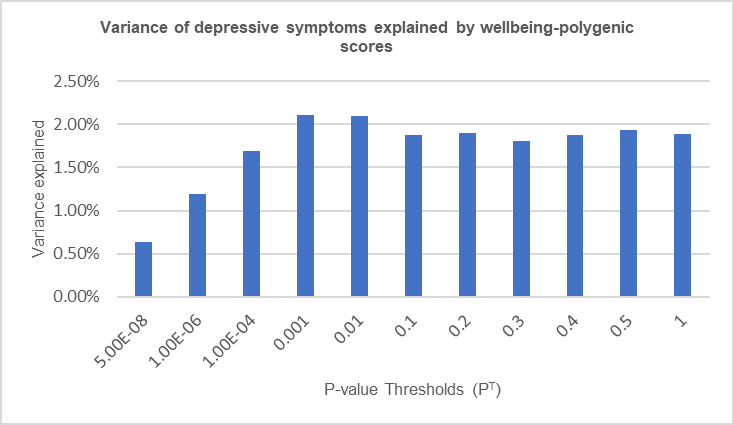

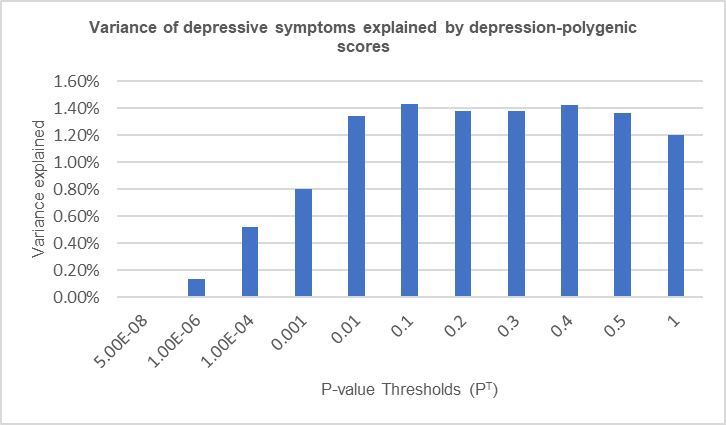

Supplement: Supplementary file 1 — Supplementary file1 (DOCX 159 kb) [file 10519_2021_10085_MOESM1_ESM.docx]
